# Effects of astaxanthin on gut microbiota of polo ponies during deconditioning and reconditioning periods

**DOI:** 10.14814/phy2.16051

**Published:** 2024-05-29

**Authors:** Mia Y. Kawaida, Kendra R. Maas, Timothy E. Moore, Amanda S. Reiter, Nicole M. Tillquist, Sarah A. Reed

**Affiliations:** ^1^ Department of Animal Science University of Connecticut Storrs Connecticut USA; ^2^ Microbial Analysis, Resources, and Services University of Connecticut Storrs Connecticut USA; ^3^ Statistical Consulting Services, Center for Open Research Resources and Equipment University of Connecticut Storrs Connecticut USA

**Keywords:** astaxanthin, deconditioning, gut microbiota, horse, reconditioning

## Abstract

To determine the effects of astaxanthin (ASTX) supplementation on the equine gut microbiota during a deconditioning–reconditioning cycle, 12 polo ponies were assigned to a control (CON; *n* = 6) or supplemented (ASTX; 75 mg ASTX daily orally; *n* = 6) group. All horses underwent a 16‐week deconditioning period, with no forced exercise, followed by a 16‐week reconditioning program where physical activity gradually increased. Fecal samples were obtained at the beginning of the study (Baseline), after deconditioning (PostDecon), after reconditioning (PostRecon), and 16 weeks after the cessation of ASTX supplementation (Washout). Following DNA extraction from fecal samples, v4 of 16S was amplified and sequenced to determine operational taxonomic unit tables and α‐diversity and β‐diversity indices. The total number of observed species was greater at Baseline than PostDecon, PostRecon, and Washout (*p* ≤ 0.02). A main effect of ASTX (*p* = 0.01) and timepoint (*p* = 0.01) was observed on β‐diversity, yet the variability of timepoint was greater (13%) than ASTX (6%), indicating a greater effect of timepoint than ASTX. Deconditioning and reconditioning periods affected the abundance of the *Bacteroidetes* and *Fibrobacteres* phyla. Physical activity and ASTX supplementation affect the equine gut microbiome, yet conditioning status may have a greater impact.

## 1 INTRODUCTION

Involved in the metabolism and immunity of the host, gut microbiota and its metabolites are key components of animal health. In the equine digestive tract, microbes ferment forages and generate short chain fatty acids (SCFA), such as acetate, butyrate, and propionate (Fernandes et al., 2014). Containing fewer than six carbons (He et al., 2020), SCFAs are essential for energy production (Costa et al., 2012) and maintenance of the intestinal barrier to prevent intestinal inflammation (Żak‐Bochenek et al., 2022). In horses, SCFAs account for approximately 65% of energy production (Costa & Weese, 2012). Disturbances of the microbiota or dysbiosis can alter fermentation patterns, which may disrupt energy utilization and contribute to the development of metabolic diseases including laminitis (Dougal et al., 2013; Kauter et al., 2019). Remaining a leading cause of critical illness in horses, gut dysbiosis can also result in gastrointestinal (GI) diseases, such as colitis, diarrhea, and gastric ulcers (Tavenner et al., 2020). Hence, sustaining the optimal gut microbial community is critical to modulate intestinal function and the overall health of animals, especially in equine athletes. Importantly, the microbial community begins and develops in prenatal and postnatal periods, respectively. While the maternal microbiome builds a foundational microbial community in offspring (Costa et al., 2016; Mols et al., 2020), microbiota composition over time is modified in its diversity and richness by several factors, such as age (Ottman et al., 2012), diet (Salem et al., 2018), and environment (Schoster et al., 2016). Notably, gut microbial responses to dietary changes such as intake of supplements have been well studied in various animal models, yet the knowledge of microbial modifications or modification periods in response to a cessation of those dietary changes is limited.

Exercise training (conditioning) is another stimulus affecting the gut microbiota composition (Mailing et al., 2019). Trained individuals often demonstrate greater bacterial diversity, abundance of SCFA‐producing taxa, and fecal SCFA concentration compared with sedentary mice (Allen et al., 2015; Evans et al., 2014; Liu et al., 2017), rats (Matsumoto et al., 2008; Mika et al., 2015), and humans (Allen et al., 2018; Barton et al., 2018; Bressa et al., 2017). Notably, mice treated with antibiotics demonstrate not only depletion in gut microbial diversity and fecal and plasma SCFA concentrations but also decreased time to exhaustion (Okamoto et al., 2019). Providing an additional substrate for exercising skeletal muscle, SCFAs support energy demands during exercise (Sales & Reimer, 2023). Therefore, greater SCFA concentration may improve endurance capacity. In horses, conditioning increases the *Bacteroidetes* phylum and decreases the *Proteobacteria* and *Spirochaetes* phyla (Janabi et al., 2016). As one of the SCFA producers, a greater abundance of *Bacteroidetes* is associated with greater energy production (Żak‐Bochenek et al., 2022). Additionally, an overabundance of *Proteobacteria* is associated with inflammatory intestinal diseases and dysbiosis, such as colic in horses (Kauter et al., 2019) and inflammatory bowel disease in humans (Costa et al., 2016). Thus, conditioning‐induced modulation of gut microbiota may provide protective effects from intestinal inflammation.

Athletes often experience deconditioning, a period characterized by reduced physical activity, due to postseason break, injury, illness, or other factors (Mujika & Padilla, 2000). Indicated by a decrease in aerobic (Koundourakis et al., 2014; Madsen et al., 1993) or anaerobic (Fatouros et al., 2005; Joo, 2016) capacity, deconditioning abolishes conditioning‐induced physiological adaptations and compromises exercise performance (Fatouros et al., 2004; Gram et al., 2015; Lawler et al., 2003). However, despite the expanding research on the impacts of conditioning on the gut microbiota composition, microbial adaptations to deconditioning, especially long‐term deconditioning remain unstudied across species. As previously discussed, gut microbiota is essential for equine health by regulating energy metabolism and the immune system (Venable et al., 2016). Understanding the response of equine gut microbiota to a period of deconditioning may allow for prevention of gastrointestinal diseases that may affect performance horses during the off‐season.

Astaxanthin (ASTX) is a natural β‐carotene found in microalgae and yeast that is often used as a feed additive for shrimp and salmon to promote pigmentation and growth. With antioxidant and anti‐inflammatory properties, ASTX contributes to not only circulating oxidative status (Baralic et al., 2013; Park et al., 2010) but also intestinal protection (Akduman et al., 2022; Kim et al., 2005; Lin et al., 2022). Astaxanthin also preserves intestinal integrity by modulating gut microbiota. Specifically, supplementation of ASTX increases *Lactobacillus* and *Bifidobacteria* at the genus level and promotes mucus secretion in the intestinal content and ileum of immunodeficient mice, respectively (Dempsey & Corr, 2022; Engevik et al., 2019; Zhang et al., 2020). In response to a high‐fat (Wang et al., 2019) or ethanol‐containing (Liu et al., 2018) diet, ASTX increases the content of *Aakkermansia* at the phylum and genus levels, respectively, resulting in thicker intestinal mucosa (Wang et al., 2019). These ASTX‐induced adaptations enhance intestinal barrier function together with the epithelial tight junctions. Establishing a link between gut microbiota, exercise, and ASTX may help develop interventions to mitigate exercise‐induced gastric inflammation, leading to enhanced athletic performance. Thus, the objective of this study was to determine the effects of ASTX supplementation on equine microbiota composition before and after exercise during deconditioning and reconditioning in polo ponies. We hypothesized that (1) deconditioning would decrease the diversity and richness of a microbial community while ASTX supplementation would mitigate these decreases, and (2) reconditioning would support a more rich and diverse microbial community while ASTX supplementation would further enhance the community.

## 2 MATERIALS AND METHODS

### 2.1 Animals

This study was reviewed and approved by the Institutional Animal Care and Use Committee (IACUC) at the University of Connecticut (A19‐056). Twelve polo ponies (10 mares and 2 geldings; *n* = 12) with a mean age of 14.8 ± 1.7 year and body weight (BW) of 494.3 ± 35.0 kg were used in the study. Horses were individually housed in the University of Connecticut's Lorentzon Stables.

### 2.2 Deconditioning, reconditioning, and washout periods

Prior to the study, all horses were actively participating in an exercise program consisting of 60 min polo fundamental lessons 4 days per week. At the start of the study, horses began a 16 week deconditioning program (Figure S1). During this period, horses received dry lot turnout approximately 4 h per day, 5 days per week, and no forced exercise. After the deconditioning period, horses underwent a 16 week reconditioning period during which time they received dry lot turnout approximately 4 h per day, 5 days per week, and completed a progressive exercise training program 4 days per week (Table S1). The last 16 weeks of the study consisted of a washout period, where the horses participated in 60 min of exercise 4 days per week to maintain their fitness status from reconditioning.

### 2.3 Diet treatment

Horses were blocked by BW, age, and sex and were randomly assigned to either a control group which received no dietary supplement (CON; *n* = 6) or a treatment group supplemented with 75 mg (approximately 0.15 mg/BW) astaxanthin (ASTX; *n* = 6; Algalíf Iceland ehf., Reykjanesbaer, Iceland) daily orally. The ASTX supplement contained no less than 10% astaxanthin oleoresin extracted from *Haematococcus pluvialis*, no more than 0.8% D‐α tocopherol, no more than 0.2% rosemary extract, and 0%–30% high oleic sunflower oil. The supplementation period started a week after the baseline exercise test and lasted for 32 weeks, during the deconditioning and reconditioning periods. Astaxanthin was mixed with 15 g ProElite Senior concentrate (Cargill Inc., Wayzata, MN) to increase palatability. Control horses received 15 g ProElite Senior concentrate (Cargill Inc.) without ASTX. Horses were individually fed twice daily with 0.3% BW/d ProElite Senior concentrate (Cargill Inc.) and 2% BW/d timothy grass mix hay. The basal diet was formulated to meet the requirement for mature horses (NRC, 2007). During the washout period, the horses remained on the same diet with no ASTX supplementation. Horses were provided with ad libitum access to a mineral salt block and water. Composited hay and grain samples were analyzed for nutrient composition at the Dairy One Forage Laboratory (Ithaca, NY) using standard, wet‐chemistry procedure (Supplementary Table 2).

### 2.4 DNA sequencing

Fresh fecal samples (three per horse) were obtained at the beginning of the study (Baseline) and after the deconditioning (PostDecon), reconditioning (PostRecon), and washout (Washout) periods. Up to 100 g of feces were collected from the center of fecal balls to avoid collecting fecal material touching the ground or other surfaces. Fecal samples were stored at −80°C until further use. Total DNA was extracted from approximately 300 mg (100 mg from each fecal ball) of fecal sample using DNeasy PowerSoil Pro Kits (Qiagen, Hilden, Germany) according to the manufacturer's protocol. Library preparation was performed at the UConn Microbial Analysis, Resources, and Services (MARS). Quantification and amplification of extracted DNA was performed as described by Chen et al. (2021). Briefly, the V4 regions of the 16S rRNA gene were amplified with Earth Microbiome version of 515F and 806R primers (Apprill et al., 2015) with Illumina adapters and dual indices. To overcome inhibition from host DNA, 0.1 pmol primer without the indexes or adapters was added to the master mix. The PCR reaction was incubated at 95°C for 3.5 min, then 30 cycles of 30 s at 95°C, 30 s at 50°C and 90 s at 72°C, followed by final extension at 72°C for 10 min. The PCR products were pooled for quantification and visualization using the QIAxcel DNA Fast Analysis (Qiagen). PCR products were normalized based on the concentration of DNA from 250 to 400 bp then pooled using the epMotion 3075 liquid handling robot. The pooled PCR products were cleaned using Omega Bio‐Tek Mag‐Bind Beads (Omega Bio‐tek, Inc., Norcross, GA) according to the manufacturer's protocol using 0.8× beads to PCR product. The cleaned pool was sequenced on the MiSeq using v2 2 × 250 base pair kit (Illumina, Inc., San Diego, CA).

### 2.5 Statistical analysis

Clinical data, operational taxonomic unit (OTU) tables, α‐diversity, and β‐diversity index were analyzed using the software package Mothur (version 1.48.0) and imported into R 4.2.1 for statistical analysis. After demultiplexing and quality checking steps, the sequences were clustered at 97% similarity. Alpha and beta diversity statistics were calculated by subsampling to 10,000 reads per sample. The Shannon diversity, Simpson's indices, and the total number of observed species (richness) were determined for the α‐diversity index. Effects of diet (CON and ASTX) and collection timepoint (TP; after deconditioning [PostDecon], after reconditioning [PostRecon], and washout) and the interaction were determined using two‐way ANOVA, followed by Tukey's test. The Bray–Curtis dissimilarity index was calculated to present β‐diversity of the bacterial community. Permutational multivariate analysis (PERMANOVA, adonis function, 99 permutations) was performed to analyze effects of diet and TP and the interaction.

Further, a Bayesian generalized linear mixed effects model was used to determine the effects of TP on OTU abundance using the method described by Sweeny et al. (2023). The analysis was done on the most abundant taxa (maximum absolute abundance >100, that is. any OTU that was at least 1% of any sample was part of “most abundant taxa”). Horse, OTU, and TP were included as a series of random effects. Models were fit using MCMCglmm (Hadfield, 2010) using a poisson family. Variance partitioning was assessed following Nakagawa and Schielzeth (2013) and code from Sweeny et al. (2023). Models were fit to all of the most abundant OTUs, as well as separately for each of the three most abundant phyla (*Spirochaete*, *Firmicutes*, and *Bacteroidetes*). Estimates of variance were then used to conduct differential abundance analyses and identify the effects of deconditioning and reconditioning on the abundance of different phyla. Baseline OTU abundance was considered a fixed effect covariate as ASTX supplementation started after the first fecal collection. Sex, age, and BW were also treated as fixed effect covariates. Analyzed results were summarized at the phylum level. Significant differences were determined at *p* ≤ 0.05.

## 3 RESULTS

### 3.1 Alpha‐ and beta‐diversity analysis

**FIGURE 1:**
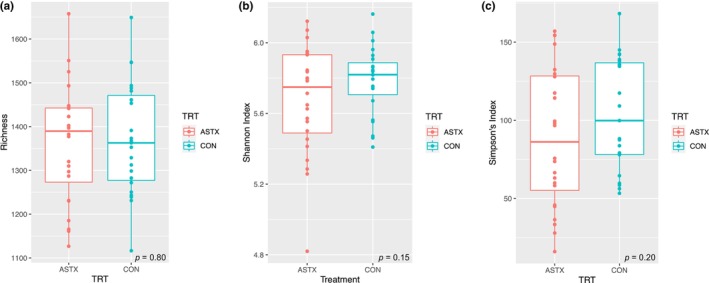
Analysis of α‐diversity. Effects of astaxanthin on richness (a) *p* = 0.80, Shannon index (b) *p* = 0.15, and Simpson's index (c) *p* = 0.20 are plotted.

**FIGURE 2:**
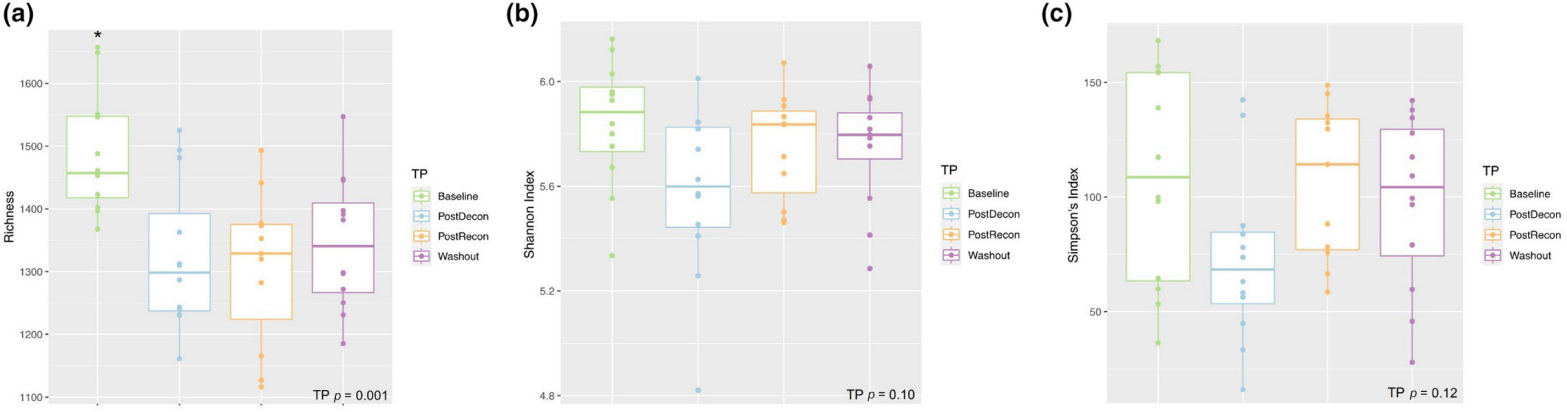
Analysis of α‐diversity. Effects of a deconditioning‐reconditioing cycle and washout period on richness (a) *p* = 0.001, Shannon index (b) *p* = 0.10, and Simpson's index (c) *P* = 0.12 are plotted. *indicates *p* < 0.05 compared with other timepoints.

Supplementation of ASTX had no effect on the total number of species (Figure 1a; *p* = 0.80) or Shannon (Figure 1b; *p* = 0.15) and Simpson's (Figure 1c; *p* = 0.20) indices. The total number of species observed in a sample was greater at Baseline than PostDecon, PostRecon and Washout (*p* ≤ 0.02; Figure 2a). The number of species remained unchanged between PostDecon, PostRecon and Washout (*p* ≥ 0.84). There were no observed effects of deconditioning, reconditioning, or washout periods on Shannon (Figure 2b; *p* = 0.10) or Simpson's (Figure 2c; *p* = 0.12) indices.

**FIGURE 3:**
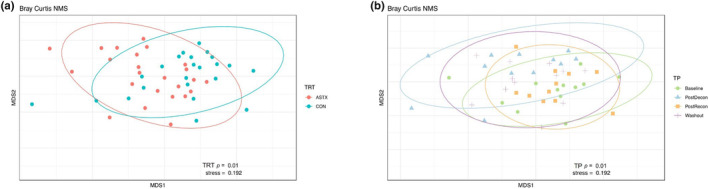
Equine gut microbiota β‐diversity in CON and ASTX (A) *p* = 0.01 and at Baseline, PostDecon, PostRecon, and Washout (B) *p* = 0.01. Metric multidimensional scaling plots represent Bray–Curtis dissimilarity indicating of samples by group. Ellipsoids represent a 95% confidence interval surrounding each group of treatment or timepoint.

A Bray–Curtis dissimilarity matrix was calculated to compare the different bacterial composition of each sample by diet and timepoint. Plotting of nonmetric multidimensional scaling (NMS) demonstrated that the communities were differentiated by ASTX (*p* = 0.01; Figure 3a), and 6% of the variability was explained by treatment. The bacterial community was also affected by timepoint (*p* = 0.01; Figure 3b) explaining 13% of the variability. There was a difference in the community between Baseline and PostDecon (*p* = 0.02) and PostDecon and PostRecon (*p* = 0.01) while Baseline, PostRecon, and Washout shared a similar microbial community (*p* ≥ 0.18).

### 3.2 Proportion of variance

**FIGURE 4:**
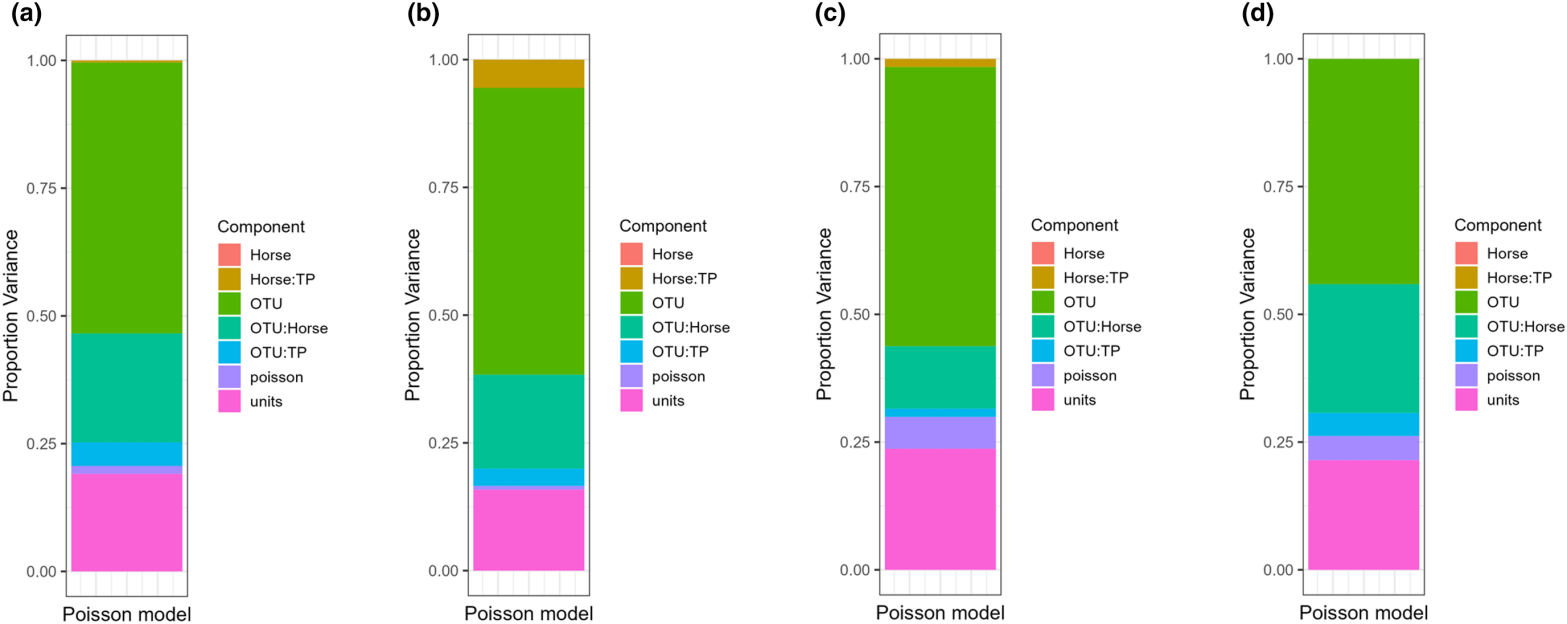
Proportion of variance in bacterial read counts from different OTUs. Comparisons across four timepoint (Baseline, Pre‐Decon, PostDecon, and Washout) were made on the overall model (a), Spirochaete (b), Firmicutes (c), and Bacteroidetes (d) at the phylum level. Plot generated using *ggregplot* (https://github.com/gfalbery/ggregplot).

While the PERMANOVA indicated significant shifts in the community based on timepoint and ASTX supplementation, the low percentage of variability explained makes it difficult methodologically to identify OTUs that correspond with those differences. Thus, we used the Sweeney method to focus on abundant OTUs (Sweeny et al., 2023). In the overall generalized linear mixed effects model, approximately 50% of variance was associated with OTUs, that is, overall variability between different OTUs in their abundance in all horses across timepoint (Figure 4a). Approximately 20% of the variance was associated with OTUs within horse, indicative of individual repeatability of OTU community composition (Sweeny et al., 2023). A moderate proportion of variance was explained by variation OTU over time (OTU:TP; 4.5%). There was less than 0.1% of variance explained by variability between horses (Horse). To further investigate how adaptations in a microbial community occur at the phylum level in response to deconditioning and reconditioning (TP), 108 of the most abundant OTUs were identified. A similar trend was observed in OTUs belonging to *Spirochaete* (Figure 4b), *Firmicutes* (Figure 4c), and *Bacteroidetes* (Figure 4d) at the phylum level where over 40% of proportion variance was associated with OTUs. Supplementation of ASTX did not affect proportion variance at the phylum level (*p* ≥ 0.53).

### 3.3 Relative abundance

**FIGURE 5:**
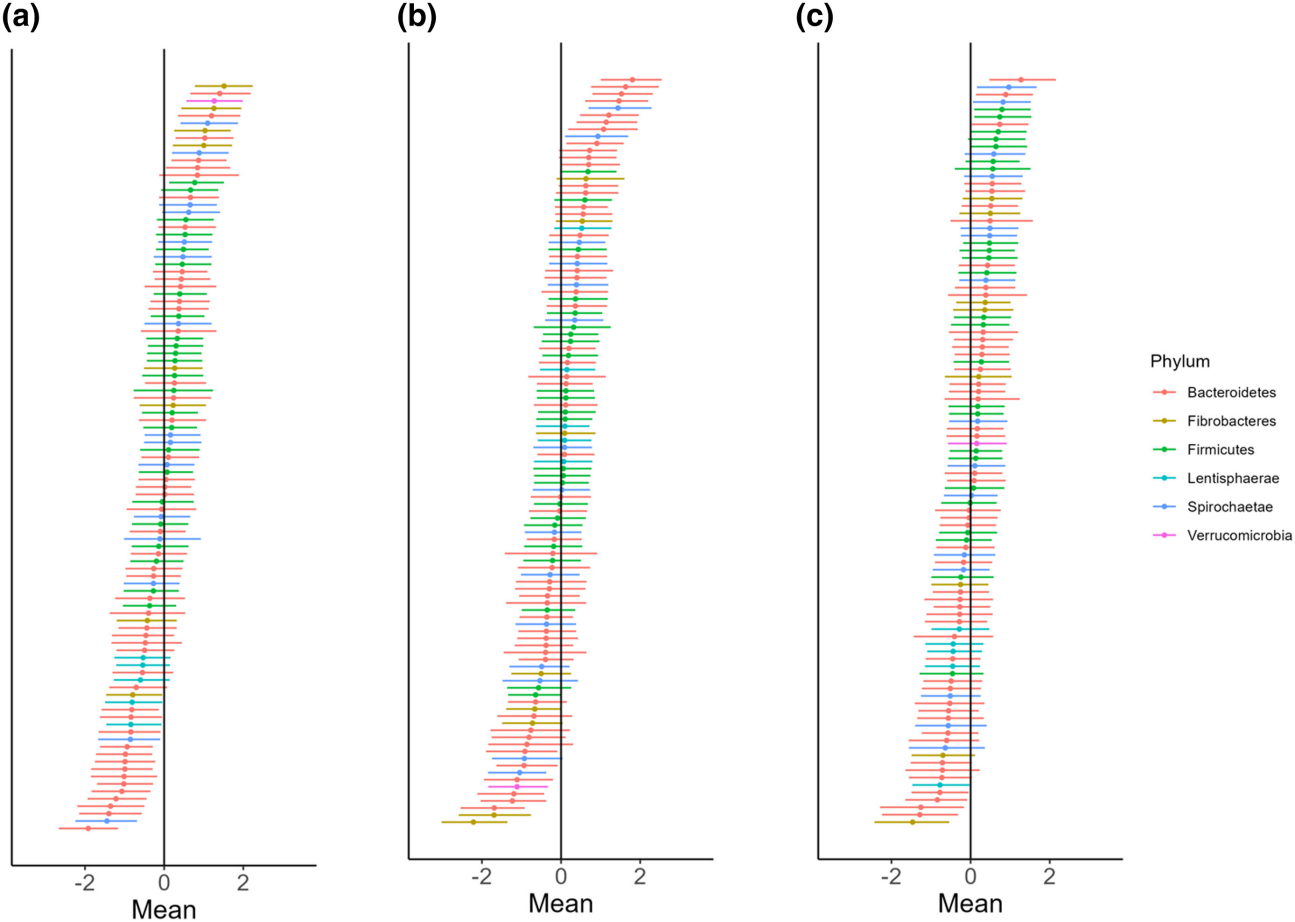
Differential abundance across different levels of deconditioning and reconditioning for the top 108 most abundant OTUs that were detected in at least 1% of samples between Baseline and PostDecon (a), PostDecon and PostRecon (b), and Baseline and PostRecon (c) are plotted. The x‐axis represents a difference from the average value of relative abundance.

There were developmental shifts in the equine gut microbiota where the abundance of *Fibrobacteres* OTUs increased following deconditioning (PostDecon) compared with Baseline while there was a decrease in the abundance of *Bacteroidetes* OTUs at the phylum level (Figure 5a). Between PostDecon and PostRecon, two of the OTUs belonging to *Fibrobacteres* experienced the greatest decrease whereas there was a greater abundance in *Bacteroidetes* OTUs (Figure 5b). The abundance of *Firmicutes* and *Spirochaetae* OTUs remained relatively consistent across timepoints. Shifts in the overall phylum abundance was the smallest between Baseline and PostRecon (Figure 5c). The mean and 95% CIs for each of the OTU used in each comparison are represented in Tables [Link], [Link].

## 4 DISCUSSION

Chronic exercise or conditioning has been reported to modulate the gut microbiota composition of rodents (Evans et al., 2014; Matsumoto et al., 2008), men (Allen et al., 2018; Mailing et al., 2019), and horses (Janabi et al., 2016). Further, intake of ASTX as a dietary supplement has been reported to increase some bacterial classes including *Bacteroidia* and *Clostridia* in the murine gut (Pratap et al., 2022). To our knowledge, this is the first study to investigate the effects of ASTX supplementation during deconditioning and reconditioning periods on the equine gut microbiota. Our results show there may be greater effects of a deconditioning–reconditioning cycle than ASTX on a microbial community.

In the present study, there was no observed effect of ASTX on α‐diversity. To our knowledge, this is the first attempt to supplement ASTX to healthy animals, especially horses, to determine gut microbial adaptations. In other studies, ASTX was supplemented to immunodeficient mice (Zhang et al., 2020) or mice with high‐fat (Wang et al., 2019) or ethanol‐containing (Liu et al., 2018) diet. While ASTX had a positive impact on α‐diversity in these mice, it is important to note that none of these studies determined the effects of ASTX on the gut microbial community in healthy individuals. We also observed a deconditioning‐induced decrease in species richness of the equine gut microbiota. Horses rely heavily on fermentation of forages for energy production (Costa & Weese, 2012). Due to the necessity of intestinal microbiota in nutrient digestion and thereby energy balance (Garber et al., 2020), reduced richness of the microbial community following deconditioning may restrict endurance capacity via impaired energy homeostasis. Interestingly, reconditioning did not reestablish the richness of the microbiome. In fact, richness following reconditioning was less than baseline. Prior to deconditioning, the horses had participated in an exercise program for 9 months, which is more than twice as long as our reconditioning program. This may indicate that adaptation of the gut microbiota richness to increased physical activity following a prolonged period of physical inactivity may require longer than 16 weeks. Gut microbial adaptations in response to regular exercise have been established in humans (Quiroga et al., 2020), mice (Yang et al., 2021), and horses (de Almeida et al., 2016). However, the effects of a deconditioning‐reconditioning cycle on the gut microbial community of different animal species, including equine athletes have not yet been investigated. Thus, further studies are required to elucidate the adaptation period of the equine gut microbiota to reconditioning following deconditioning as competitive horses are likely to experience a period of reduced physical activity due to post‐season breaks, injuries, or diseases.

Additionally, relative abundance of taxa was not impacted by ASTX at any timepoint. Contrary to our finding, a previous report demonstrated that supplementation of 30, 60, or 120 mg/kg BW ASTX ameliorated a decrease in abundance of *Lactobacillus* and *Bifidobacteria* in immunodeficient mice in a dose‐dependent manner (Zhang et al., 2020). However, when 1 mg/day ASTX was given to healthy mice, the abundance of *Bacteroidia* and *Clostridia* increased (Pratap et al., 2022). The conflict between the results of the present study and previous work may be explained by a difference in the health status of animals, supplement dose, and/or animal model used in the studies. In this work, healthy horses with no apparent GI issues received approximately 0.15 mg/BW ASTX mixed with a concentrate feed. As a fat‐soluble material, the absorption of ASTX increases when it is given with lipid‐rich substance or food (Meor Mohd Affandi et al., 2012). ProElite Senior concentrate, which contains a minimum of 10% crude fat (CF), was used to mix ASTX in to increase palatability. The CF content of Senior concentrate is greater than that of balanced concentrate, which is often fed to young equine athletes. However, use of a high‐fat supplement such as flaxseed might improve ASTX absorption and thereby its effects. Altogether, it may be important to consider individual's health, GI conditions, dosage, and/or how the ASTX is delivered when using ASTX as a nutrition supplement to induce a richer microbial community.

Deconditioning and reconditioning periods affected relative abundance of the *Bacteroidetes* phylum in this study. A greater abundance of *Bacteroidetes* has been reported in lean or aerobically trained humans (Morita et al., 2023; Motiani et al., 2020) and animals (Evans et al., 2014). Correspondingly, we observed a decrease and an increase in some of the *Bacteroidetes* OTUs following deconditioning and reconditioning, respectively. Some *Bacteroidetes* contribute to energy generation by producing SCFA, including acetate and propionate (Besten et al., 2013). As prolonged exercise requires greater energy production and expenditure, having a greater abundance of *Bacteroidetes* may improve exercise endurance capacity. Morita et al. (2023) have demonstrated supplementing mice with *Bacteroidetes uniformis* during a conditioning period extended swimming time to exhaustion along with an increase in acetate and propionate. Altogether, an abundance of *Bacteroidetes* in feces may be an adequate indicator of physical capacity. Moreover, horses suffering from GI diseases have lesser abundance of *Bacteroidetes* than healthy horses (Lara et al., 2022). Deconditioning, therefore, may increase the prevalence of GI diseases via a reduction of *Bacteroidetes* whereas regular exercise may have a reverse effect.

In conclusion, ASTX may impact β‐diversity regardless of deconditioning and reconditioning periods in equine athletes. Moreover, the equine gut microbiota composition was significantly altered by a deconditioning‐reconditioning cycle. Further investigations are required to assess whether these modifications are consistent in response to acute strenuous exercise and whether a greater dose of ASTX may increase the magnitude of the changes observed in the present study. Elucidation of an interaction between antioxidant supplementation and deconditioning and reconditioning periods may help develop interventions to enhance energy homeostasis during prolonged exercise.

## AUTHOR CONTRIBUTIONS

M.Y.K. and S.A.R. conceived and designed research; M.Y.K., A.S.R., and S.A.R. performed experiments; M.Y.K., K.R.M., T.E.M. analyzed data; M.Y.K. interpreted results of experiments; M.Y.K. prepared figures; M.Y.K. drafted manuscript; M.Y.K., K.R.M., T.E.M., A.S.R., N.M.T., and S.A.R. edited and revised manuscript; M.Y.K., K.R.M., T.E.M., A.S.R., N.M.T., and S.A.R. approved final version of manuscript.

## FUNDING INFORMATION

This study was funded by a University of Connecticut Research Excellence Program grant to S. A. Reed.

## CONFLICT OF INTEREST STATEMENT

No conflicts of interests, financial or otherwise, are declared by the authors.

## ETHICS STATEMENT

This study was reviewed and approved by the Institutional Animal Care and Use Committee (IACUC) at the University of Connecticut (A19‐056).

## Supporting information


Data S1.

Table S3.

Table S4.

Table S5.

## Data Availability

The data that support the findings of this study are available from the corresponding author upon reasonable request.
