# Supplementary material for: Effects of astaxanthin on gut microbiota of polo ponies during deconditioning and reconditioning periods
Source: Physiol Rep. 2024 May 29;12(11):e16051. doi: 10.14814/phy2.16051 (PMC11136553; doi:10.14814/phy2.16051)
Supplement: Supplementary file 1 — Data S1. [file PHY2-12-e16051-s004.docx]

SUPPLEMENTAL MATERIAL

Supplementary Figure 1. Study timeline. The horses underwent a 16-wk deconditioning and reconditioning periods, during which the treatment group received daily oral supplementation of 75mg astaxanthin (ASTX). Deconditioning was followed by a 16-wk washout period, where the ASTX supplementation was discontinued.

Supplementary Table 1. 16-wk reconditioning program. Intensity and duration of exercise gradually increased as the program progressed.

| Week | Walk (min) | Trot (min) | Canter (min) |
| --- | --- | --- | --- |
| 1 – 3 | 30 | 10 | - |
| 4 – 5 | 20 | 20 | 3 |
| 6 – 7 | 20 | 30 | 10 |
| 8 – 16 | 15 | 20 | 15 |

**Supplementary Table 2.** Nutrient composition^1^.

| Nutrient^2^ | ProElite Senior Concentrate | Timothy Grass Mix Hay | |
| --- | --- | --- | --- |
| DE, Mcal/kg | 3.08 | 2.01 | |
| CP, % | 17.8 | 10.8 | |
| ADF, % | 20.3 | 41.3 | |
| NDF, % | 30.4 | 64.1 | |
| Ca, % | 1.31 | 0.36 | |
| P, % | 0.65 | 0.22 | |
| Mg, % | 0.38 | 0.19 | |
| K, % | 1.32 | 2.05 | |
| Na, % | 0.51 | 0.03 | |
| Fe, ppm | 519 | 235 | |
| Zn, ppm | 305 | 19.0 | |
| Cu, ppm | 57.0 | 6.00 | |
| ^1^Nutrient composition was analyzed by Dairy One, Inc. (Ithaca, NY).  ^2^All nutrients are presented on a 100% dry matter basis. | | |  |
